# Supplementary material for: Viral infection drives cell-intrinsic re-localization of the C. elegans immune-repressive STAT transcription factor STA-1
Source: bioRxiv. 2026 Jun 29:2026.06.24.734234. Preprint. [Version 1] doi: 10.64898/2026.06.24.734234 (PMC13344970; doi:10.64898/2026.06.24.734234)

Supporting Information

Fig. S1 DRH-1(2CARD) protein interactors identified by co-IP/MS

bioRxiv preprint doi: https://doi.org/10.64898/2026.06.24.734234; this version posted June 29, 2026. The copyright holder for this preprint (which was not certified by peer review) is the author/funder, who has granted bioRxiv a license to display the preprint in perpetuity. It is made available under a CC-BY 4.0 International license.

(A) Differentially enriched proteins when comparing integrated DRH-1(2CARD) (*jyIs37) vs.* mScarlet control (*jyIs41).* Both constructs expressed in *rde-1(ne300)* mutant background. (B) Differentially enriched proteins when comparing extrachromosomal DRH-1(2CARD) (*jyEx305) vs.* mScarlet control (*jyIs41).* Extrachromosomal DRH-1(2CARD) is expressed in a WT background. Differentially enriched proteins determined by |log2FC| > 4 and p < 0.01.

Fig. S2 Time course of changes in STA-1::GFP expression upon viral infection
Representative images of uninfected or virus-infected adult animals at 12, 24, and 48 hpi. Animals were infected at the L4 stage. Viral infection was visualized using Quasar 670-conjugated (far-red) FISH probes targeting the Orsay virus genome. Nuclei were counterstained using DAPI (blue). White arrowheads indicate nuclei; orange arrowheads indicate autofluorescence. Scale bar = 10 μm.

Fig. S3 STAT phylogenetic analysis
Phylogenetic tree constructed with amino acid sequences of full-length STAT proteins. Text shading indicates mammalian STATs and annotated homologs. Node coloring indicates taxonomic rank. Bootstrap values over 50 are listed at nodes.

Fig. S4 Foldseek identifies human STAT5a/STAT5b as top structural matches for STA-1 Alphafold predicted structure. Overlay of STA-1 Alphafold structure (white) and top 6 structural matches: STAT5a (pink/purple), STAT5b structures (blue/green), and STAT6 (yellow). Lower E-values indicate higher similarity between structures. Root mean square deviation (RMSD) is reported for all atoms in both structures ($RMSD_{all}$) or for atoms at aligned amino acid residues (RMSDaligned).

Fig. S5 STA-1 negatively regulates genes induced by viral RNA1 replicon and infection. (A) WormCat analysis of upregulated genes in *sta-1* vs. control RNAi in *virEx26[RNA1(mt)]* background. (B) Volcano plot of differentially expressed genes (DEGs) in *virEx23[RNA1(WT)]* vs. *virEx26[RNA1(mt)]*. Both groups were treated with control RNAi. Upregulated (green) and downregulated (magenta) genes determined by |log2FC| > 0.6 and p < 0.05. Labels indicate top 20 upregulated genes. (C) WormCat analysis of upregulated genes in *virEx23[RNA1(WT)]* vs. *virEx26[RNA1(mt)]* animals treated with control RNAi. For (A) and (C) gene counts and p-values are displayed in scaled bubble charts. Enrichment scores were determined using a false discovery rate cutoff of 0.01. (D) Genes upregulated by both *sta-1* RNAi and *virEx23[RNA1(WT)].* (E) Genes

upregulated by both *sta-1* RNAi and DRH-1(2CARD). (F) The proportion of STA-1-regulated genes (green) ranges from 0 to 0.14 of total upregulated virus response genes at a given timepoint during the infection time course (Castiglioni et al. 2024).

Supplementary Tables

S1 Table. *C. elegans* strains used in this study

S2 Table. DNA constructs used in this study

S3 Table. Primers used in this study

S4 Table. Co-immunoprecipitation mass spectrometry results

S5 Table. Differentially expressed genes in *virEx23* L4440 vs *virEx26* L4440

S6 Table. Differentially expressed genes in *virEx23 sta-1* RNAi vs *virEx23* L4440

S7 Table. Differentially expressed genes in *virEx23 sta-1* RNAi vs *virEx26 sta-1* RNAi

S8 Table. Differentially expressed genes in *virEx26 sta-1* RNAi vs *virEx26* L4440

S9 Table. Differentially expressed genes in *jyIs37* vs *jyIs41*

FIG S1

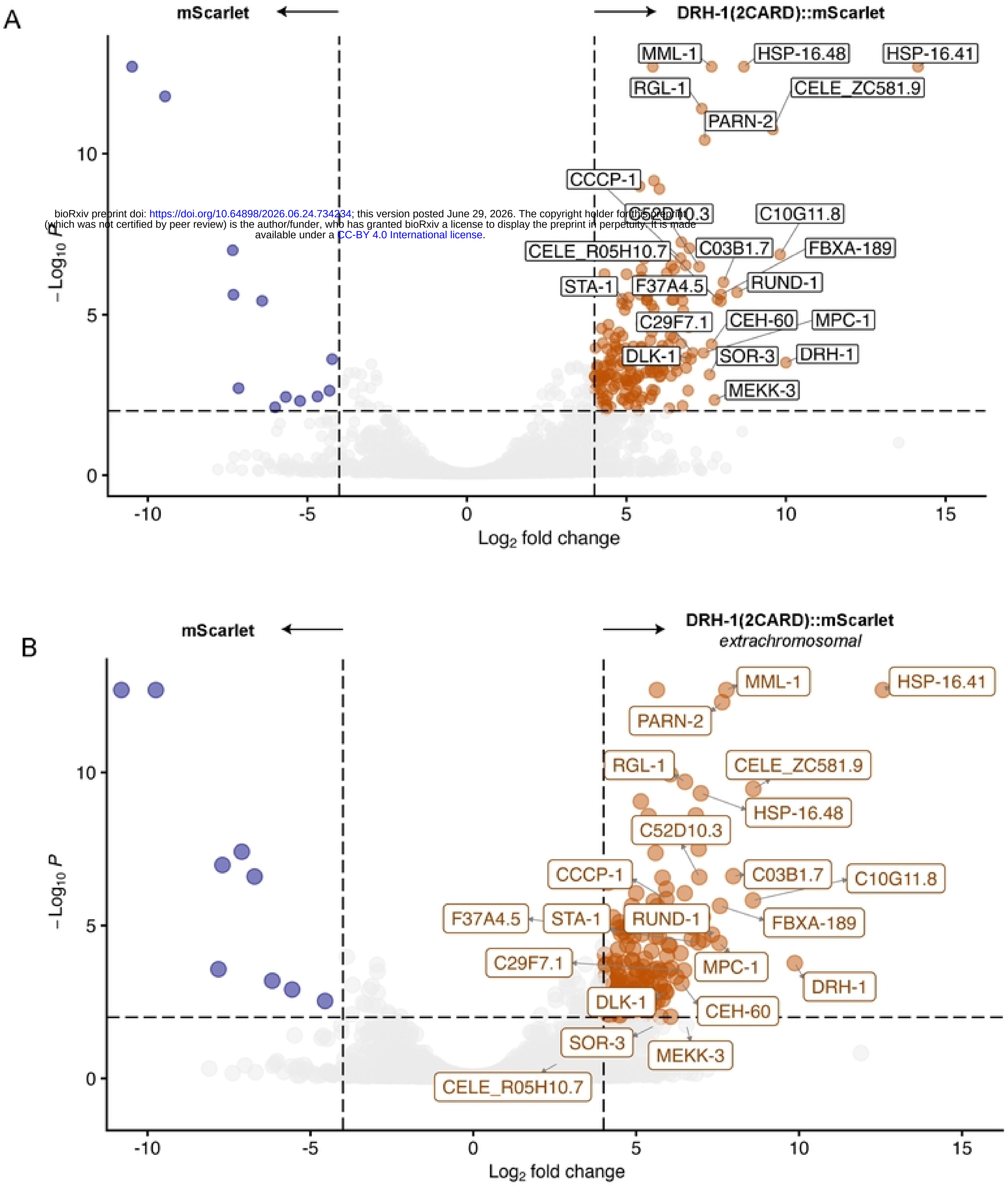

bioRxiv preprint doi: https://doi.org/10.64898/2026.06.24.734234; this version posted June 29, 2026. The copyright holder for this preprint (which was not certified by peer review) is the author/funder, who has granted bioRxiv a license to display the preprint in perpetuity. It is made available under a CC-BY 4.0 International license.

FIG S2

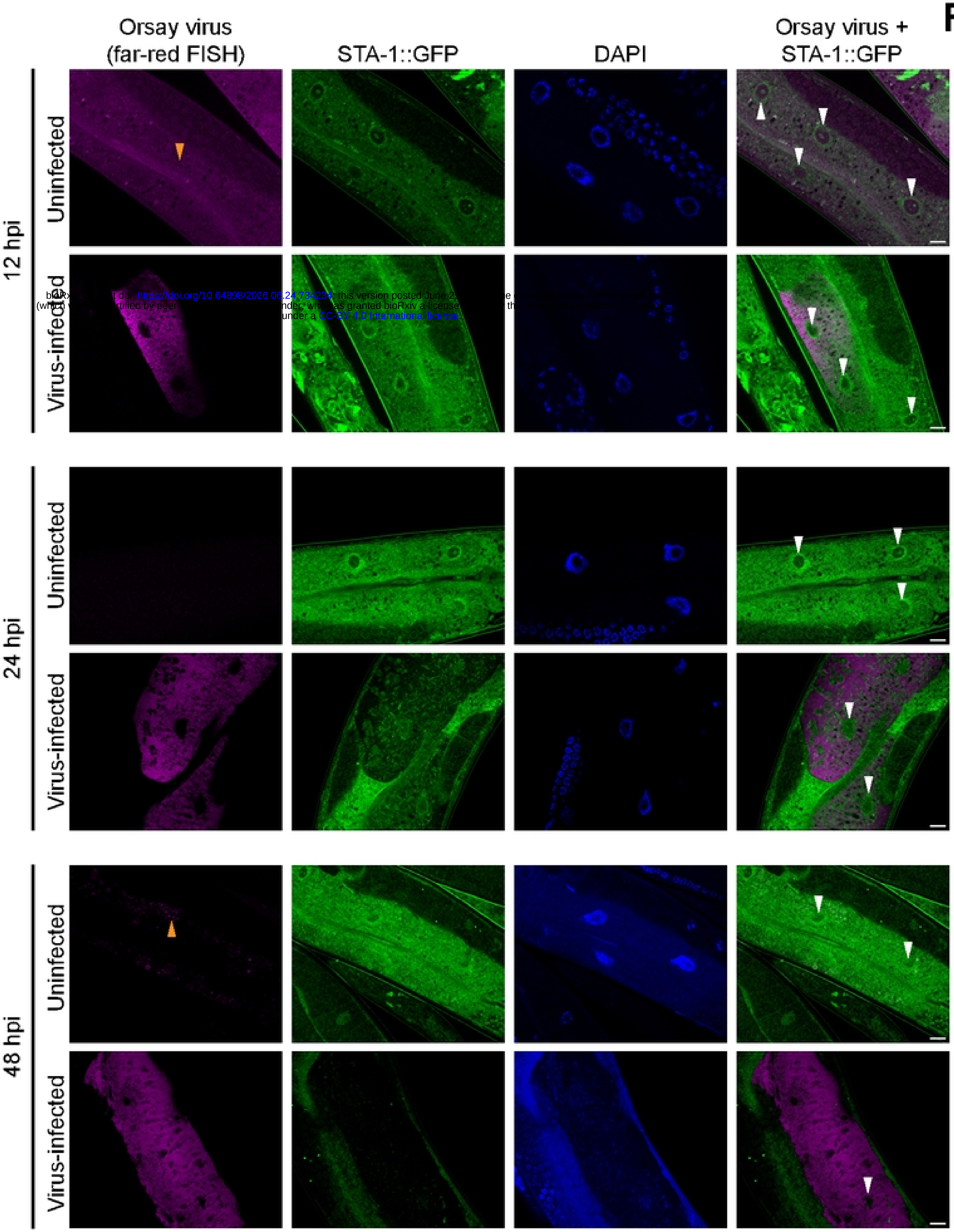

bioRx[illegible]t doi: https://doi.org/10.64898/2026.06.24.734234; this version posted June 2[illegible]e [illegible]
(which [illegible]rtified by peer [illegible]nder, wh[illegible]as granted bioRxiv a license [illegible] th[illegible]
under a CC-BY 4.0 International license.

bioRxiv preprint doi: https://doi.org/10.64898/2026.06.24.734234; this version posted June 29, 2026. The copyright holder for this preprint (which was not certified by peer review) is the author/funder, who has granted bioRxiv a license to display the preprint in perpetuity. It is made available under a CC-BY 4.0 International license.

FIG S3

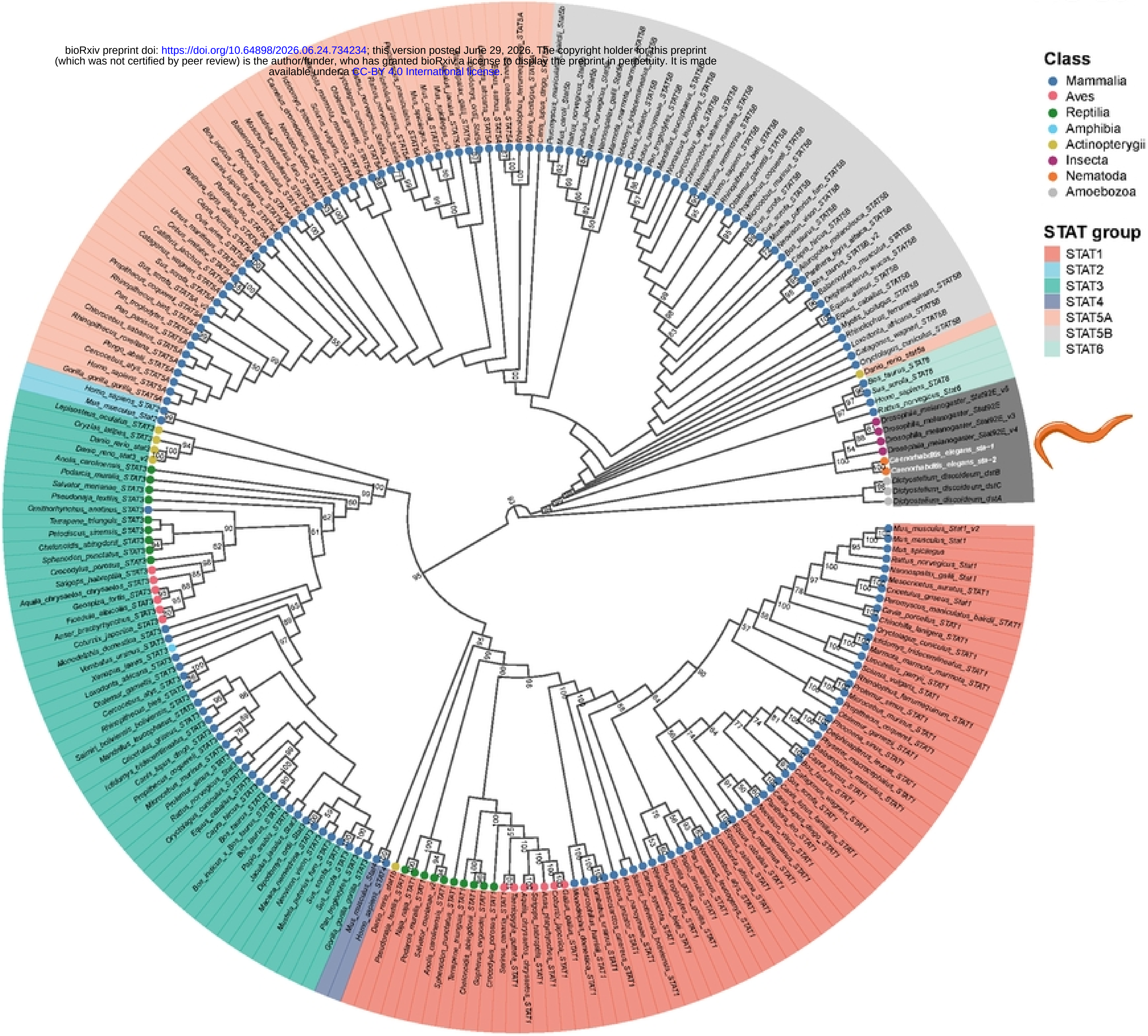

FIG S4

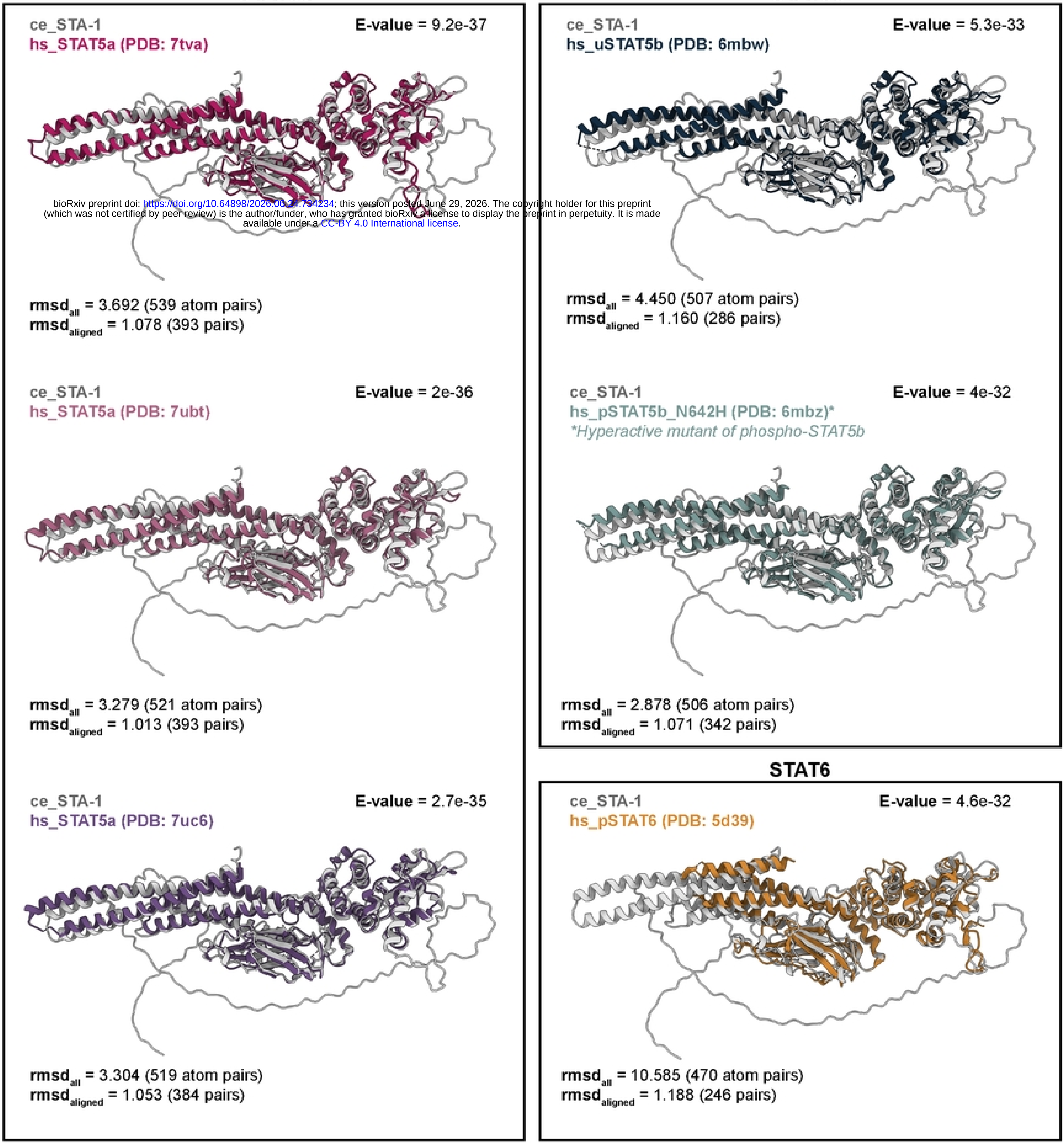

bioRxiv preprint doi: https://doi.org/10.64898/2026.06.24.734234; this version posted June 29, 2026. The copyright holder for this preprint (which was not certified by peer review) is the author/funder, who has granted bioRxiv a license to display the preprint in perpetuity. It is made available under a CC-BY 4.0 International license.

FIG S5

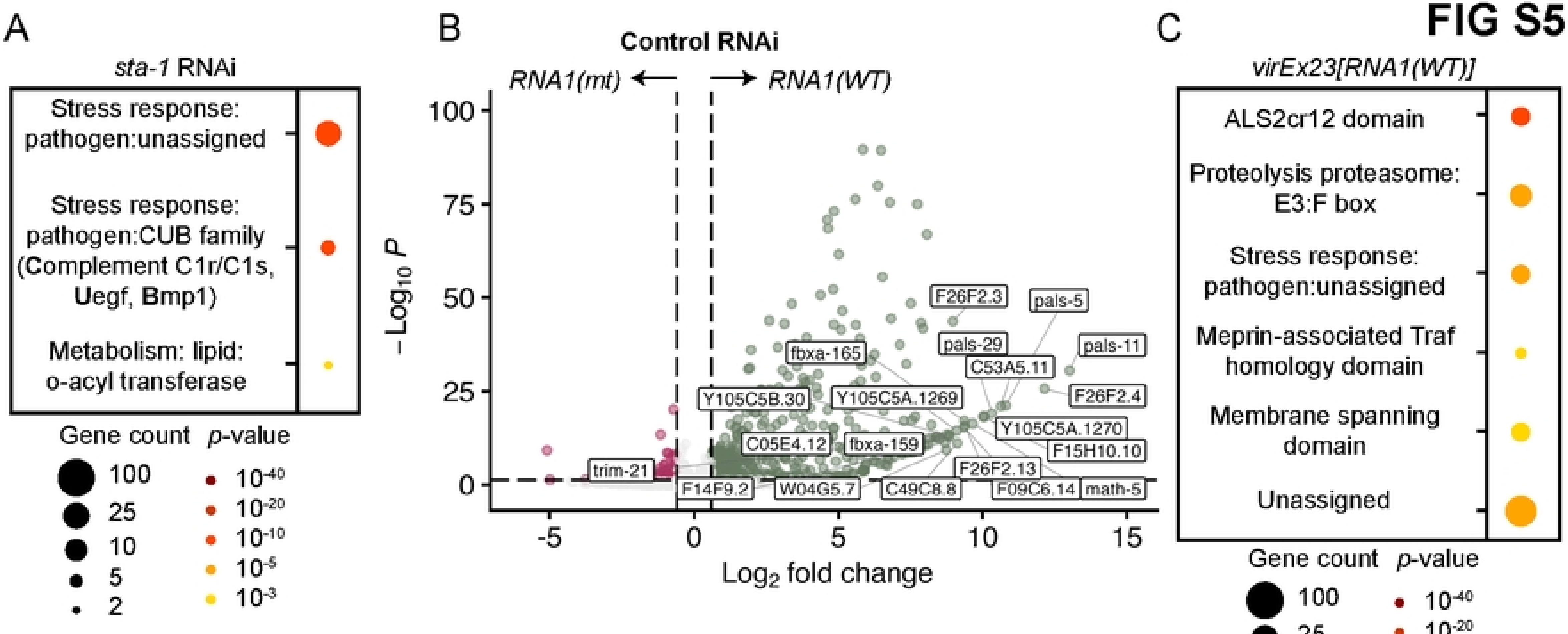

D

Upregulated by *sta-1* RNAi and *virEx23[RNA1(WT)]*		
ZK355.8	*F23D12.1*	*F53B2.8*
K10H10.9	*B0348.2*	*fbxa-164*
tsp-2	*bath-46*	*F40F12.9*
pals-36	***F15B9.6***	*ncx-8*
ZK896.1	*C08E8.4*	*F47D12.6*
tsp-1	*warf-1*	*ZC239.14*
fbxa-88	*zip-5*	*oac-5*
Y47H10A.5	***skr-5***	*fbxc-58*
Y26D4A.3	*T15B7.10*	*valv-1*
B0024.4	*sdz-6*	*oac-57*
F33H12.7	***Y46G5A.20***	*F19B2.5*
cgt-1	*T19D12.4*	*C41G7.8*
clc-19	***Y37H2A.14***	*arrd-7*
K10D11.3	*C49C3.9*	*math-37*
C49G7.7	*K09D9.1*	***dod-23***

E

Upregulated by *sta-1* RNAi and DRH-1(2CARD)	
T19C9.8	*T15B7.10*
pals-36	***Y46G5A.20***
ZK896.1	***Y37H2A.14***
Y47H10A.5	*C49C3.9*
Y26D4A.3	*F53B2.8*
F33H12.7	*fbxa-164*
B0348.2	*bath-47*
bath-46	*ZC239.14*
arrd-3	*F19B2.5*
F15B9.6	*bath-26*
skr-5	*math-37*

F

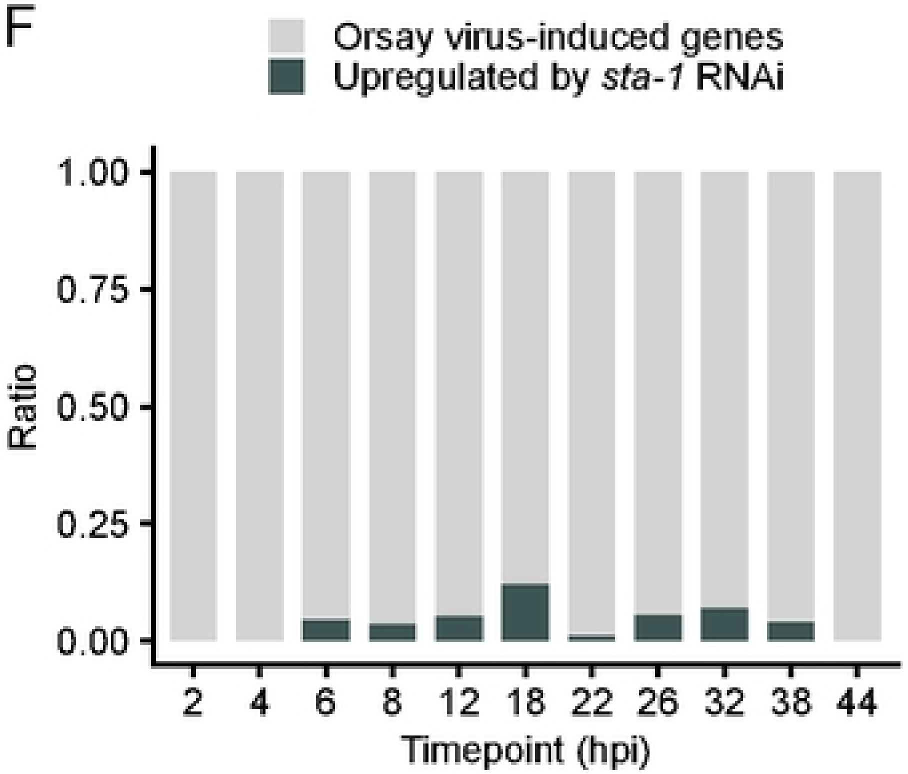

Supplement: Supplement 1 [file NIHPP2026.06.24.734234v1-supplement-1.pdf]
